# An Overview of In Vitro Biological Neural Networks for Robot Intelligence

**DOI:** 10.34133/cbsystems.0001

**Published:** 2023-01-10

**Authors:** Zhe Chen, Qian Liang, Zihou Wei, Xie Chen, Qing Shi, Zhiqiang Yu, Tao Sun

**Affiliations:** ^1^ School of Medical Technology, Beijing Institute of Technology, Beijing 100081, China.; ^2^ Key Laboratory of Biomimetic Robots and Systems (Beijing Institute of Technology), Ministry of Education, Beijing 10081, China.; ^3^ Advanced Innovation Center for Intelligent Robots and Systems, Beijing Institute of Technology, Beijing 100081, China.; ^4^ School of Mechatronical Engineering, Beijing Institute of Technology, Beijing 100081, China.

## Abstract

In vitro biological neural networks (BNNs) interconnected with robots, so-called BNN-based neurorobotic systems, can interact with the external world, so that they can present some preliminary intelligent behaviors, including learning, memory, robot control, etc. This work aims to provide a comprehensive overview of the intelligent behaviors presented by the BNN-based neurorobotic systems, with a particular focus on those related to robot intelligence. In this work, we first introduce the necessary biological background to understand the 2 characteristics of the BNNs: nonlinear computing capacity and network plasticity. Then, we describe the typical architecture of the BNN-based neurorobotic systems and outline the mainstream techniques to realize such an architecture from 2 aspects: from robots to BNNs and from BNNs to robots. Next, we separate the intelligent behaviors into 2 parts according to whether they rely solely on the computing capacity (computing capacity-dependent) or depend also on the network plasticity (network plasticity-dependent), which are then expounded respectively, with a focus on those related to the realization of robot intelligence. Finally, the development trends and challenges of the BNN-based neurorobotic systems are discussed.

## Introduction

Artificial neural networks (ANNs) have made great strides in recent years, pushing artificial intelligence to surpass the human brain in many specific tasks, such as Go [1], video games [2], diagnosis [3], etc. Despite this, when it comes to more general and more abstract tasks requiring adaptive learning and cognition, the human brain still shows better performance [4]. With the help of the brain, humans can perceive, understand, adapt to, and actively change their environment. The brain is intrinsically a biological neural network (BNN) composed of tens of billions of biological neurons connected with each other by structures called synapses [5]. Compared with ANNs, BNNs still hold advantages in many aspects: higher energy efficiency, parallel computing, real-time learning, self-healing, autonomous consciousness (active cognition), etc. [6–9]. These superior performances of BNNs largely benefit from the nature of biological neurons and BNNs in information processing [10]. A neuron, the basic unit of a BNN, processes information by firing action potentials (APs; or spikes) according to its membrane potential (the difference between the internal and external potentials of the neuron) through an “all-or-none” principle: When its membrane potential exceeds the firing threshold from below, the neuron fires an AP. In addition, a synapse is a bridge connecting 2 neurons, which enables the AP emitted by the presynaptic neuron to cause a change in the membrane potential of the postsynaptic neuron, so that the signal is transmitted from the presynaptic neuron to the postsynaptic neuron. Moreover, the strength of synapses adaptively changes in response to incoming signals and postsynaptic neuron responses, which are thought to underlie the brain’s ability to learn and store memories [5].

Traditional ANNs are essentially computational models that imitate these characteristics of BNNs, but their computing efficiency and energy efficiency are far inferior to BNNs [6]. The operation of the human brain only consumes about 20 W of power, while running an ANN for autonomous driving can easily exceed several kilowatts of power [11]. To further imitate biological neurons and BNNs to optimize ANNs, researchers proposed neuromorphic computing [12] and spiking neural networks (SNNs) [13,14]. While neuromorphic computing mimics biological neurons at the hardware level, SNNs mimic the BNNs at the computational level [15]. They have shown superiorities in diverse perspectives compared with conventinal ANNs, further promoting the development of artificial intelligence [16]. Therefore, increasing understanding of BNNs has definitely inspired and promoted the progress of artificial intelligence [17]. Moreover, the biological intelligence possessed by BNNs in itself may be directly used to control robots, thereby endowing the robots with intelligence. Some thoughts on whether consciousness or intelligence could arise in robots endowed with BNNs and their potential effects and ethical issues can be found in the study of Warwick [18].

Research has shown that, other than in the brain, BNNs can also exist independently from biological bodies [19,20]. The isolated primary neurons can still connect with each other to form a BNN in a Petri dish, and the above-mentioned functional properties of neurons and synapses are preserved [21,22]. This holds the potential for culturing neurons in a Petri dish to construct an in vitro BNN and then to realize biological intelligence in vitro. For example, in vitro BNNs can exhibit some preliminary intelligent behaviors, including stimulus-dependent synaptic plasticity [23], supervised learning [24,25], input-dependent adaptation [26], associative memory [27], logical operation [28], short-term memory [29], spatiotemporal memory [30], blind source separation (unsupervised learning) [23,31], homeostatic plasticity [32,33], etc. Although the in vitro BNNs may exhibit a certain degree of intelligence, as a pure information processing device, like the brain, it cannot directly interact with the environment in itself. The brain interacts with the environment through the body, which receives sensory stimuli from the external environment and transmits them to the brain, and the brain sends movement instructions to the body to act on the environment [34]. In order to interact with the environment, the in vitro BNN also needs a “body” that can perceive and act on the environment in real time. A robot is naturally suitable to be such a “body”. The BNN can be bidirectionally connected to the robot in this way: The sensor output of the robot is encoded as the input of the BNN, and the output of the BNN is decoded as the driver input of the robot. Thereby, the in vitro BNN can directly control the robot to perform specific tasks, such as avoiding obstacles, playing video games, finding targets, etc. [35–43]. Although much progress has been made in this field, a comprehensive review of in vitro BNNs for robot intelligence is still missing.

Therefore, in this review, we aim to provide a comprehensive overview of the intelligent behaviors exhibited by in vitro BNNs, with a particular focus on those related to robot intelligence. Our overall goal is to reduce the knowledge barriers for researchers and practitioners in the field of artificial intelligence and robotics to understand the intelligent behaviors of BNNs, thereby promoting the development of research using in vitro BNNs for robot control or robot intelligence. Compared with other existing reviews [11,18,44], which share at least a part of the topics involved in this review, this review features a focus on BNNs ([11] focusing on SNNs), more technical details and comprehensive consideration of related work (compared with [18]), intelligent behaviors exhibited by BNNs (even without robots), and the mechanisms underlining the intelligent behaviors (compared with [44]).

**Fig. 1.**
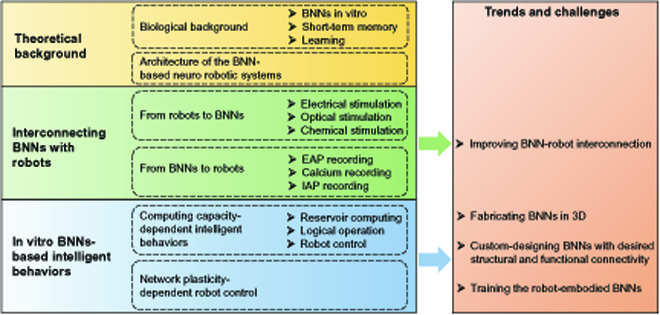
The outline of this review.

The remainder of this review is organized as follows. In Theoretical Background, we introduce the relevant biological background of BNNs and the architecture of the BNN-based neurorobotic systems. In Methods for Connecting BNNs with Robots Bidirectionally, we outline the technical means to interconnect in vitro BNNs with robots. In In Vitro BNN-based Intelligent Behaviors, we divide the intelligent behavior based on in vitro BNNs into 2 categories: computing capacity-dependent and network plasticity-dependent intelligent behaviors, with a special focus on those related to robot control. We summarize the development trends and challenges in Trends and Challenges and conclude this review in Conclusion. The outline of this review is shown in Fig. 1.

## Theoretical Background

### Biological background

#### In vitro BNNs

**Fig. 2.**
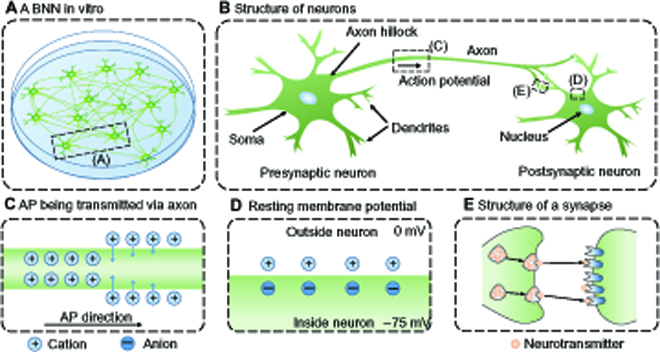
Structural diagrams of BNNs, neurons, and synapses. (A) A biological neural network (BNN) cultured in a Petri dish. (B) Enlarged view of two neurons connected with each other. Further zoom-in pictures of an action potential (AP) being transmitted via the axon (C), resting membrane potential in a small area of the postsynaptic neuron (D), and the structure of a synapse (E).

All the BNNs in nature, from the brain of *Caenorhabditis elegans*, which contains only a few hundred neurons [45], to the human brain containing 86 billion neurons [46], form under a self-organization way: Neurons connect with each other naturally through structural connections called “synapses”. A neuron consists of 3 parts: the soma, the dendrites, and the axon, which share the same cell membrane. Structural diagrams of BNNs, neurons, and synapses can be shown in Fig. 2. In general, because of the difference in ion concentration inside and outside the membrane, the potential inside the membrane is slightly lower than that outside the membrane. This difference in potential, called membrane potential, is about −75 mV when it receives no input. The axon of one neuron (presynaptic neuron) can form synapses with the dendrites or soma of another neuron (postsynaptic neuron). The synapses are a structure that propagates signals unidirectionally in an “electrical-chemical-electrical” manner. More biological background knowledge of BNNs can be found elsewhere [47]. In short, compared to other cells, neurons have the following special mechanisms: (a) When the membrane potential of its cell body reach a certain threshold from below, an AP will be launched along the axon (start from the axon hillock), and when the AP signal reaches the synapses, the synapses will release neurotransmitters to the postsynaptic neurons; (b) when the small membrane area at soma or dendrites of a postsynaptic neuron receives the neurotransmitter released by the synapse, the membrane potential changes at that location [called postsynaptic potential (PSP)], and this PSP signal can passively propagate along the cell membrane; and (c) the strength of a synapse, that is, the amount of neurotransmitter released at a single time, changes with the firing activity of the pre- and postsynaptic neurons, resulting in the change of PSPs. On one hand, the first 2 mechanisms endow the biological neurons with nonlinear computing capacity, that is, they can integrate complex spatiotemporal information and emit signals in an “all or none” manner [48]. Moreover, complex network structures in BNNs further strengthen their nonlinear computing capacity. On the other hand, on the basis of the third mechanism (synaptic plasticity), neurons can encode and store complex neural spatiotemporal information. Synaptic plasticity of all neurons in a BNN as a whole is referred to as network plasticity instead, which is considered to underlie the mechanisms of memory and learning in the BNN [49].

These mechanisms of neurons can be preserved in nonphysiological Petri dish environments. In some early studies, neurons from embryonic or newborn mouse brains were isolated and seeded in Petri dishes, which could develop into mature neurons, and synapses could be formed between neurons, thus forming BNNs [50,51]. The nonlinear computing capacity and network plasticity of these BNNs closely resemble those in the brain [33]. Therefore, building BNNs in Petri dishes has gradually become an important branch of neuroscience. However, in contrast to its in vivo counterpart (the brain), which can interact with the environment through the body and then shows various intelligent behaviors, the in vitro BNN lacks such a body to interact with the environment. The introduction of the multielectrode array (MEA) changed this situation [52]. The MEA can simultaneously record from and act on the in vitro BNNs [53], which played an important role in exploring the nonlinear computing capacity and network plasticity of in vitro BNNs [29,30]. Moreover, the MEA can serve as a stable interface to connect an in vitro BNN with a computer for a long time. By using the computer, a virtual robot can be simulated to interact with a virtual environment. Then, the sensory signal of the robot is fed back to the BNN, and its neural spatiotemporal activities are monitored and decoded to control the robot so that the BNN can interact with the virtual environment through the virtual robot [39]. Other studies took a further step by connecting the in vitro BNN with a real robot via the computer, thus allowing the BNN to interact with the real environment [35,36,38,54]. In this review, such a system that interconnects an in vitro BNN with a robot is called a BNN-based neurorobotic system.

#### Short-term memory

Dranias et al. [29] found that the in vitro BNN exhibited short-term memory ability, which refers to the ability to store small amounts of stimulus-specific information. This study found that the in vitro BNN, like the BNN in the brain, has 2 memory processes for storing short-term information: the fading and the hidden memory process. Among them, the fading memory stores information through sustained firing activity evoked by the stimulus-evoked rebound in the network. Different stimuli can elicit distinguishable sustained neural activities that last up to 1 s. Hidden memory process relies on synaptic plasticity to encode incoming signals to changes in synaptic strength in the network. By applying additional stimuli, it was observed that the response activity evoked by the stimulus changed after the input signal was applied, so that the input information was preserved in the network. This part of memory was stored longer and can survive the loss of neural activity until it was erased by the next occurrence of a network-wide burst. These results suggested that in vitro BNNs have the same short-term memory mechanism as BNNs in the brain [55]. In a follow-up study, Ju et al. [30] also found that this short-term memory capability can be used to store complex spatiotemporal stimulus information. In these studies, spontaneous neural activity in the in vitro BNNs, typically network-wide bursts, had a negative effect on the memory process. Therefore, inhibiting these activities may enhance the memory capacity of BNNs, especially the hidden memory capacity [56]. Research in these directions may bring new insights into the understanding of memory processes in the brain and may also open up new areas of biological storage.

#### Learning

Theoretically, memory is a prerequisite for learning. However, the ability of in vitro BNNs to learn requires 2 other prerequisites: The number of synaptic connections is large, and the connections are stable [24]. Shahaf and Marom [24] demonstrated that, on the basis of these 3 conditions, the in vitro BNN can perform supervised learning through closed-loop training. In this study, BNNs were cultured on an MEA and interacted with a computer-controlled virtual environment by EAP recording and electrical stimulation through the MEA’s electrodes. Through closed-loop training, it was demonstrated that this BNN has the ability to learn and memorize random tasks (specific firing patterns). In short, applying a fixed input stimulus to the BNN in the dish can induce random neural firing patterns. One of the firing patterns was selected as the pattern expected to be induced by the BNN. In closed-loop training, stimulation is stopped whenever the desired pattern occurs. This training process is closed-loop because the monitored signal of the recording electrodes is analyzed and fed back to control the stimulation signal. The time to elicit the desired pattern decreases with increasing training time, indicating that through closed-loop training (a supervised learning strategy), the BNN learns to exhibit the desired neural firing pattern for this input stimulus. In a follow-up research, Feber et al. [25] repeated the work of Shahaf and Marom [24] and took a special look at the induced functional connectivity changes in the BNN. They had 2 main findings. First, they found that stimulation at fixed electrodes can induce network connectivity changes of a similar magnitude compared with stimulation at randomly varied electrodes. Second, slow electrical stimulation (the same as in the work of Shafaf and Marom) applied through a closed-loop way can force the network to converge to a different balance state, compared with open-loop stimulation. In another follow-up research, Li et al. [57] chose a similar training protocol with 2 modifications. First, voltage stimulation mode was used instead of current stimulation mode. Second, biphasic pulses with positive phase first were used instead of those with negative phase first. However, the BNN can still be trained to present spontaneous synchronized bursts with increased synchrony (stable balance state) under such a training protocol. This proved that a wider choice of stimuli may be used for supervised learning on BNNs. Other than slow stimulation, Hamilton et al. [58] demonstrated that high-frequency stimulation can also be used to train the BNN to present increased response duration evoked by the same probe signal.

Other studies have shown that in vitro BNNs can perform unsupervised learning. Tanaka et al. [59] found that the BNN in the dish can exhibit response specificity to different spatial pattern stimuli and can also enhance its response specificity after repeated exposure to a pattern stimulus (unsupervised training). Isomura et al. [23] found that an in vitro BNN can perform blind source separation, an unsupervised learning process, which refers to the process of extracting independent source signals from mixed signals. They further confirmed that the in vitro BNN can perform blind signal separation because BNN inherently has a tendency to minimize its variational free energy, which results in the process of blind source separation [31]. That is to say, the in vitro BNNs perform unsupervised learning based on the free energy theory [60].

### Architecture of the BNN-based neurorobotic systems

**Fig. 3.**
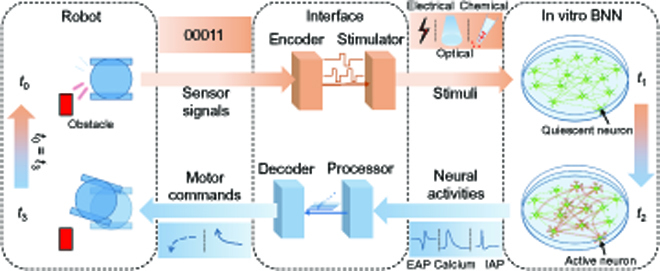
The schematic architecture of the BNN-based neurorobotic systems.

The architecture of a typical BNN-based neurorobotic system is shown in Fig. 3. The system consists of 3 parts: an in vitro BNN, an interface, and a robot. Among them, the interface is a general term for all devices interconnecting the BNN and the robot, including computers (processors, encoders, and decoders), recording devices (omitted in Fig. 3), and stimulation devices (stimulators). The BNN interacts with the environment through the robot as the body, which accepts the stimuli from the environment, and actively acts on the environment, while the BNN plays the role of the brain. Normally, this BNN–environment interaction can be seen as being composed of many continuous and independent basic feedback loops, each of which can be divided into 2 processes: from the robot to the BNN and from the BNN to the robot. During the former process, the robot accepts stimuli from the environment at time *t*_0_ and converts them into sensory signals, which are encoded and act on the in vitro BNN at time *t*_1_> through a stimulator, causing changes in neural activity in the BNN at time *t*_2_. During the latter process, the neural activity in the BNN is recorded, processed, and then decoded into the motor commands and sent to the robot; then, the robot moves according to the received commands at time *t*_3_. Each basic feedback loop experiences a delay (*t*_3_ − *t*_0_) from sensation (*t*_0_) to movement (*t*_3_), also known as the reaction time. Every 2 basic feedback loops are interlocked by setting the end time (*t*_3_) of the previous basic feedback loop as the start time (*t*_0_) of the next one so that the BNN can continuously interact with the environment. In another way more resembling the brain–environment interaction, these 2 processes are seen as independent from each other and thereby are run not in sequence, but in parallel. In this case, system delay can be greatly reduced, therefore allowing real-time BNN–environment interaction.

## Methods for Connecting BNNs with Robots Bidirectionally

To enable the in vitro BNNs to interact with the environment through the robots, the BNNs must be able to communicate with the robots bidirectionally. As mentioned before, this bidirectional communication consists of 2 unidirectional processes: from robots to BNNs and from BNNs to robots.

### From robots to BNNs

**Fig. 4.**
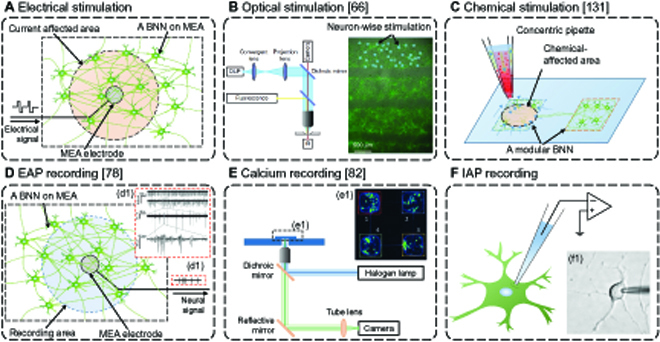
Illustration and examples of the mainstream stimulation and recording methods for in vitro BNNs. (A) In electrical stimulation, electrical signals are transmitted to an electrode, which stimulates a small number of neurons near this electrode. (B) With optical stimulation, neuron-wise stimulation can be achieved. (C) Chemical stimulation can apply a local stimulation both spatially and temporally, with flexible scalability. (D) In EAP recording, neuronal activity around a specific electrode is recorded as a sum. (E) In calcium recording, higher spatiotemporal resolution can be achieved. (F) In IAP recording, the highest temporal resolution can be achieved. All pictures are adapted with permission from the references indicated above under the terms of the CC BY 4.0 license. BNN, biological neural network; MEA, multielectrode array; DLP, digital light projector; IR, infrared light; EAP, extracellular action potential; IAP, intracellular action potential.

In the process from robots to BNNs, the sensor signals of the robots are first encoded to stimulus signals, which are then transmitted to the BNNs through the stimulator, thereby converting the stimulus signals into neural signals. In these 2 signal transmissions, the latter one of converting stimulus signals (digital signals) into neural activity (biological analog signals) is very challenging and under intense research [61]. The current mainstream stimulation methods for delivering digital signals to in vitro BNNs include the following 3 categories: electrical stimulation, optical stimulation, and chemical stimulation. Schematics and examples of them are shown in Fig. 4.

#### Electrical stimulation

Negative electrical signals can be applied to the extracellular solution through electrodes, pulling down the extracellular potential of neurons, thereby increasing their membrane potential, and possibly causing it to fire APs. MEAs are commonly used devices for applying electrical stimulation to in vitro BNNs, which consists of multiple microelectrodes arranged in an array on a flat surface [53]. These conductive microelectrodes are connected to stimulators by nonconductive leads [62]. The stimulators generate electrical signals, usually charge-balanced biphasic waveforms [35–43,63], which are transmitted to the microelectrodes via the lead and induce a drop in the extracellular potential of neurons near the microelectrodes, thereby inducing the neurons to generate APs.

In addition to the MEA, bipolar electrodes can also be used to apply electrical stimulation to the in vitro BNN [64]. Compared to the MEA, bipolar electrodes can apply local extracellular stimulation in a larger area of stimulation and in a more flexible manner, but the number of channels that can simultaneously apply stimulation is limited [65]. The patch–clamp technique, which uses a micropipette to interact with the neuron, can also be used to apply electrical stimulation in a more precise manner [66]. It can induce potential changes outside a small patch of membranes using the cell-attached mode (the micropipette attached to the neuron) [67]. Alternatively, it can induce the intracellular potential to vary using the whole-cell mode (the micropipette attached to the neuron and then break the membrane by suction) [68]. However, this method suffers from a limited number of stimulus channels and cannot last for too long because of disturbances and damages to the neuron.

#### Optical stimulation

Neurons do not have photo-sensitive channels and therefore do not respond to optical stimulation. However, after optogenetic treatment [69], the photosensitive channel protein will be expressed on the neuronal membrane, which can produce channel-specific changes to particular cations under light exposure with a specific range of wavelength, resulting in the influx of the cations and the increase in membrane potential, which, in turn, induces AP [70]. Therefore, after optogenetic treatment of the in vitro BNNs, a digital micromirror-based projector equipped with a special light path can be used to achieve precise optical stimulation at single-neuron [30,33,66] or even subneuron resolution [71]. A recent research combined optogenetics and MEA to achieve a closed-loop system between in vitro BNNs and a computer [72], which paved the way for further robot control.

In addition to optogenetics, caged compounds, which are light-sensitive chemicals that can be activated by light exposure, can also be used for optically stimulating in vitro BNNs [73]. For example, when RuBi-glutamate, a kind of caged glutamate, is added to the culture medium, irradiation liberates the trapped glutamate, resulting in increased BNN excitability [43]. By changing the light intensity and irradiation time, the concentration of activated glutamate in the solution can be changed, thereby changing the excitability of the BNN, so as to achieve the purpose of regulating the neural activity of the network through optical stimulation.

#### Chemical stimulation

In nature, most of the signal transmission between neurons is achieved by chemicals (neurotransmitters). Therefore, chemicals like excitatory neurotransmitters (glutamate) can also be applied to in vitro BNNs to induce neural activities [28,74]. In addition to neurotransmitters, high-concentration potassium solutions are commonly used for chemical stimulation by elevating the membrane potential [75,76].

According to their area of stimulation, chemical stimulation can be divided into local stimulation and full-field stimulation. By diffusing the applied drugs across the entire culture medium, the full-field stimulation acts on the entire BNN [75]. Local stimulation requires the help of some special equipments, such as separate microchambers [76], concentric pipettes [28,74,77], etc., to constrain the chemical stimulation in a small area.

### From BNNs to robots

In the process from BNNs to robots, the neural activities (biological analog signals) of the BNNs are recorded as digital signals, which are then processed into neural spike sequences, decoded into motion control signals, and sent to the robots to guide their motion. Among them, the key step is the recording of neural activity. Current mainstream methods for recording the neural activity of in vitro BNNs are extracellular AP (EAP) recording, calcium recording, and intracellular AP (IAP) recording, schematic illustrations of which are shown in Fig. 4.

#### EAP recording

The neural activity of a BNN is composed of the individual activity of each neuron within it. When a neuron fires an AP, first, sodium ions flood into the cell (causing the membrane potential to rise), then potassium ions flow to the outside of the cell (membrane potential falls), and, finally, sodium and potassium ions return to their normal level under the influence of the sodium–potassium pump so that the membrane potential gradually returns to the resting potential. During an AP, both the intra- and extracellular potentials of the cell change [5]. The methods of recording the extracellular potential of neurons and then determining the onset time of each AP by analysis are called EAP recording [50,78]. The commonly used equipment for EAP recording is the MEA [53,54]. Each microelectrode on the MEA can not only be used to actively apply electrical stimulation but also passively record the potential around it, which typically consists of a superposition of the activity of multiple surrounding neurons. Through band-pass filtering, this potential can be divided into 2 components: the low-frequency part called local field potential and the high-frequency part multiunit activity [79]. Since the duration of an AP is very short, usually within 5 ms, the high-frequency multiunit activity signals are then used for determining the exact onset of each AP for each detectable neuron by various spike detection (or spike sorting) algorithms. Reviews on extracellular spike detection/sorting algorithms can be found elsewhere [80,81].

#### Calcium recording

An AP is also accompanied by the momentary opening of calcium ion channels so that calcium ions flood into the cell. By loading the calcium ion fluorescent indicator into the neuron [28,74,77,82] or making it express genetically encoded calcium indicators in itself [83], the change of calcium ion concentration can be converted into the change of fluorescence intensity, and this change can be observed by a fluorescence microscope. This technique is called calcium imaging, also known as calcium recording [84,85]. Compared with EAP recording, calcium recording has the following advantages: (a) higher spatial resolution; (b) the ability to map the recorded neural activity to a specific neuron; and (c) less expensive equipment requirements. However, the duration of stable recording is not as long as that of the EAP recording, which can lasts for months [86], while calcium recording normally lasts less than a few hours due to photobleaching of the calcium indicator [82].

#### IAP recording

Since the AP is accompanied by a substantial change in the intracellular potential, the neural activity of the neuron can be detected by recording the intracellular potential [87]. A means of recording intracellular membrane potential is patch clamp in the whole-cell mode [68], where the tip of the glass micropipette is attached to a neuron and breaks the membrane to allow its inner fluid to blend with the cytoplasm of the neuron. The main advantage is that the recording noise is at a minimal level, and the main disadvantages include the following: (a) The manipulation is difficult because the contact between the inner fluid of the glass microelectrode and the cytoplasm is difficult to form [88]; and (b) the spatial resolution of the recording is limited, with, currently, a maximal of 16 neurons can be recorded simultaneously [89]. From this perspective, it can hardly be used to build a long-term stable connection between BNNs and robots. However, efforts are being made to use microfluidic technics for IAP recording (called planar patch clamp) [90], which holds the potential to build a BNN–robot connection as steady as using MEAs.

Because of their shortcomings in calcium recording and IAP recording, right now they are rarely used separately in the neural activity recording of in vitro BNNs. The MEA-based EAP recordings are the most prevalent due to their inherent ability to simultaneously apply electrical stimulation. However, in recent years, many studies have begun to combine multiple recording methods to achieve better recording results [91–93]. Moreover, other novel recording methods, such as optical voltage imaging [94], are also being considered in the recording of in vitro BNNs [95].

## In vitro BNN-based Intelligent Behaviors

**Fig. 5.**
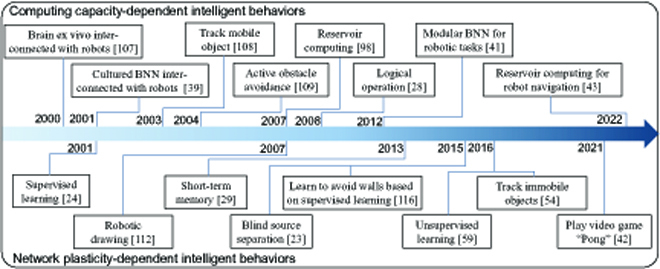
Milestones of the intelligent behaviors exhibited by in vitro BNNs or BNN-based neurorobotic systems.

The in vitro BNNs embodied by robots have the ability to interact with the environment, so they can show some preliminary intelligent behaviors. In addition, some studies have begun to mine the intelligent behaviors inherent in the in vitro BNNs. These intelligent behaviors are inseparable from the 2 basic characteristics of BNNs: computing capacity and network plasticity. Therefore, in this chapter, according to the 2 capabilities, we divide these intelligent behaviors into 2 categories: computing capacity-dependent and network plasticity-dependent. The former mainly relies solely on the nonlinear computing capacity of BNNs, while the latter relies more on their network plasticity. An overview of the milestones of intelligent behaviors exhibited in in vitro BNNs or BNN-based neurorobotic systems is shown in Fig. 5.

### Computing capacity-dependent intelligent behaviors

Since the in vitro BNNs are formed by cultured neurons building synapses with each other and the neurons are capable of receiving synaptic input and output APs in an “all or none” manner in itself, these BNNs feature nonlinear computing capacity. Part of the intelligent behaviors of in vitro BNNs relies on this computing capacity, which mainly includes 3 categories: reservoir computing, logical operation, and robot control.

#### Reservoir computing

Reservoir computing (RC) originated from recurrent neural networks, special ANNs with feedback connections, which makes them suitable for dynamic (temporal) data processing [96]. A reservoir computing system consists of an input layer, a computational reservoir, and a reader (output layer), where the computational reservoir is responsible for nonlinearly mapping the input signal to a high-dimensional space, and the reader (often simple and linear) is trained to estimate the nonlinear mapping model from the input signal to the output signal. The traditional computing reservoir is often a recurrent neural network composed of artificial neurons, but, theoretically, any physical system with high-dimensional, nonlinear computing capacity and fading memory (the influence of input on system state fades with time) such as an in vitro BNN can serve as a reservoir [97]. These RC systems based on in vitro BNNs usually use MEAs to input electrical stimuli and record neural responses.

In an earlier study, researchers demonstrated the ability of the in vitro BNN-based RC system to separate 2 stimulation patterns [98]. Subsequent studies confirmed that these RC systems could classify multiple stimulation patterns. Dockendorf et al. [99] utilized an in vitro BNN as a reservoir and employed a single electrode for stimulation input, so the spatial stimulation pattern could reach the number of stimulation electrodes on the MEA (60 in this research). The spatiotemporal spike sequence was evoked by the stimulus signal, and the mapping from this pulse sequence to the input spatial stimulus pattern was achieved using a linear classifier, thereby demonstrating the ability of the BNN-based RC system to separate 60 spatial stimulus inputs. Ortman et al. [100] further demonstrated that such a system is able to separate out a wider variety of spatiotemporal stimulation patterns. Specifically, they constructed a BNN as a liquid state machine on an MEA with 59 electrodes. Among them, 32 electrodes were used as stimulation electrodes and were divided into groups of 4 to produce a total of 8 groups. Each group of stimulating electrodes sequentially applies electrical stimulation at fixed time intervals to be a stimulation pattern. Six time intervals were chosen, resulting in a total of 48 stimulation patterns. Results showed that fewer stimulation patterns and lower stimulation frequency would lead to higher classification accuracy.

However, in these studies, the output of the BNN is mapped to another high-dimensional space by a nonlinear reader, and a nonlinear classifier is used to classify the response, so the nonlinear separability of the BNN itself has yet to be proven. The nonlinear separability of the reservoir state is the greatest advantage of reservoir computing because it allows the use of linear readers, which greatly reduces the difficulty of training. To this end, a follow-up study nonlinearly mapped the 3-dimensional (3D) spatiotemporal spike trains of BNNs induced by spatiotemporal stimulation patterns into high-dimensional (120D) spatial patterns, allowing linear separation of these spatial patterns [101]. Therefore, this study shows that the state of the in vitro BNN can be processed nonlinearly to fit a linear reader to fully exploit the advantages of reservoir computing. In other studies, instead of using MEAs to apply electrical stimulation, the researchers began to use optogenetic means and digital projectors to input optical stimulation signals. This significantly increased the spatial resolution of input stimulation to the level of a single neuron, which endows these RC systems with better input separability [29,30].

#### Logical operation

The computing capacity inherent in the BNNs can also be used to perform logical operations. In early studies, Feinerman et al. [74,77] demonstrated that patterned BNNs could reliably perform a series of logical operations, including threshold, AND gate, and diode (66% success rate), which could then be combined to implement a neuronal oscillator [28]. The fabrication of neuronal diodes, which could realize unidirectional signal propagation between neuronal populations (modules), has then become a hot topic. Although neuronal diodes could be obtained by a modified Hewlett–Packard plotter equipped with a sharp metal tip writing on cell-repellent substrates, this technique limits the neuronal patterns at the mesoscale. To fabricate neuronal diodes in a more scalable way, diverse microfluidic techniques were explored. By using microcontact printing, Albers and Offenhäusser [102] improved the success rate of the neuronal diode to 85%. By confining the cultured neurons in microfluidic chambers, Forró et al. [103] achieved a success rate of 92%, and Peyrin et al. [104] obtained a neuronal diode with a 95% success rate of unidirectional signal propagation.

However, these studies only achieved unidirectional signal propagation at population levels. Yamamoto et al. [105] demonstrated that this diode structure can be achieved between 2 neurons using microcontact printing. Yoshida et al. [106] also demonstrated the unidirectional signal propagation by first seeding neurons on micropillars and then assembling 2 neuron-anchored micropillars together. Neuronal logic devices, implemented by in vitro BNNs, with the development of biofabrication techniques for custom-designing neuronal circuits, are attracting ever-increasing attention.

#### Computing capacity-dependent robot control

**Fig. 6.**
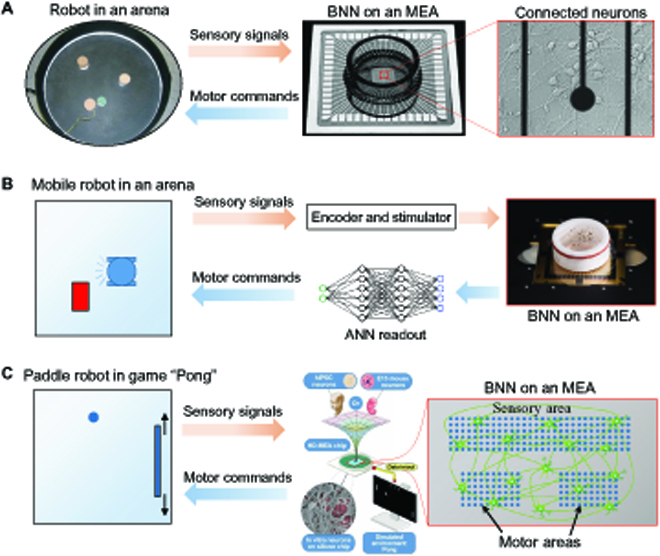
Representative studies on robots controlled by in vitro BNNs. (A) and (B) belong to the computing capacity-dependent robot control studies, where the robots rely solely on the computing capacity of BNNs to perform specific tasks, adapted with permission from the study of Novellino et al. [38] under the terms of the CC BY 3.0 license, and from the study of Aaser et al. [111] under the terms of the CC BY 4.0 license, respectively. (C) A network plasticity-dependent robot control study, where the BNN can “learn” how to improve performance, adapted with permission from the study of Kagan et al. [42] under the terms of the CC BY 4.0 license. hIPSC, human induced pluripotent stem cell; E15, embryonic day 15;

Many studies have demonstrated that in vitro BNNs can control robots to perform specific tasks, of which some representative studies are shown in Fig. 6. Some of these studies rely solely on the computing capacity of BNNs, while others rely more on the network plasticity of BNNs to perform tasks. A list of representative studies on robots controlled by in vitro BNNs is shown in Table 1. We will discuss the latter in the Network plasticity-dependent intelligent behaviors subsection, and in this subsection, we focus on the former.

**Table 1.** Representative references on computing capacity-dependent robot control.

Ref.	Recording/stimulation	Encoding/decoding	Robot tasks
[36]	EAP/electrical	Binary coding of stimulation frequency based on the front sonar sensor/proportional mapping based on the spike firing frequencies of 2 motor electrodes	Obstacle avoidance
[37]	EAP/electrical	Fixed probe stimuli/nonlinear mapping based on evoked network activity	Adaptive flight control
[38]	EAP/electrical	Binary or proportional coding of stimulation frequency based on the proximity sensor/winner-take-all for the slow wheel and proportional mapping for the fast wheel	Obstacle avoidance
[39]	EAP/electrical	Spatial coding of sensory electrodes based on the robot’s movement state/randomly allocating clustered spatiotemporal patterns to motor commands	No specific task
[41]	EAP/electrical	Proportional coding of stimulation frequency based on proximity sensors/winner-take-all for the slow wheel and proportional mapping for the fast wheel	Obstacle avoidance
[43]	EAP/electrical + optical	Binary coding of stimulation frequency based on the camera and infrared sensors/proportional mapping based on the output of a linear readout	Tracking an immobile object
[107]	EAP/electrical	Proportional coding of stimulation frequency based on the weighted sum of the light sensors/proportional mapping based on the motor populations’ firing frequency	No specific task
[108]	EAP/electrical	Proportional coding of interstimulus interval based on the distance to the object/proportional mapping based on the average network activity	Tracking a mobile object
[109]	EAP/electrical	Proportional coding of stimulation frequency based on proximity sensors/proportional mapping based on the motor populations’ firing frequency	Obstacle avoidance
[111]	EAP/electrical + chemical	Nonlinear transformation based on the proximity sensors and the output of an ANN readout/nonlinear mapping based on the output of an ANN readout.	Obstacle avoidance
[131]	Calcium/chemical	Binary coding of stimulation frequency based on the proximity sensor/binary mapping based on the motor area’s network activity	Obstacle avoidance

An early study isolated the brain of a lamprey and preserved it in a Petri dish to stimulate and record neural signals in the brain by inserting electrodes (glass micropipettes) into specific brain regions [107]. The recorded signals were decoded to control a mobile robot, while the robot’s sensor signals were encoded to determine stimulus signals, forming a closed-loop robot control loop. Isolating the intact animal brain and interconnecting it with the robot have the advantage of preserving the neural circuit of the BNN. However, this approach also faces many problems: (a) Decapitation can cause mental and physical damage to the brain; (b) even if the intact brain is isolated in Petri dish, access to its internal neurons is still difficult; (c) lack of physical support, the brain has a short survival period in a Petri dish, so it cannot support long-term research; and (d) even the lamprey brain has a relatively small number of neurons, it is difficult to cover the monitoring of its entire brain area with current neural recording methods. Therefore, subsequent studies turned to culturing isolated primary neurons in Petri dishes to form in vitro BNNs. Compared with the isolated animal intact brain, the in vitro BNNs have the following advantages: (a) The lack of obstruction of the skull and meninges makes it easier to access neurons and record neural signals; (b) longer survival period; and (c) the relatively small number of neurons makes monitoring the entire BNN possible. DeMarse et al. [39] used an MEA to realize the bidirectional connection between an in vitro BNN and a virtual robotic rat. They recorded the spontaneous neural activity of the BNN using 60 electrodes of the MEA and identified the current activity pattern through a self-learning classifier. The identified activity patterns are randomly mapped to one of 4 motion states: front, back, left, and right. This motion state will be transmitted to the robotic rat in a simulated environment to guide its motion. At the same time, the sensory signals of the robotic rat, including proprioceptive signals (4 motor states) and collision detection signals, are transmitted to the MEA and encoded into the stimulation signals of the 5 stimulation electrodes. In this way, the in-dish BNN can sense the environment (stimulated by the MEA) in real time and act on the environment (by directing the robot to move). However, the motivation for this system is to study how in vitro BNNs process and encode information for a better understanding of the brain, so, in this study, the BNN did not control the robot rat to achieve specific tasks.

In a later research, Shkolnik [108] used an in vitro BNN to control a mobile robot (called Koala) to achieve the task of tracking a random moving target (another mobile robot, called Khepera). In this study, Koala’s sensor signals are fed into the BNN via the MEA, and the MEA also monitors the BNN’s network activity and maps this activity to Koala’s control signals. Here, the BNN plays the role of an input–output mapping. Therefore, for the stability of the control, a robust input–output mapping relationship needs to be found. In this study, this relationship is the mapping from the interstimulus interval to average network activity.

Other studies exploited a more direct input–output mapping. Martinoia et al. [109] used part of the electrodes on the MEA as motor areas (divided into left and right motor areas) and 2 of the other electrodes as sensory areas (one for the left sensory area and the other for the right sensory area). The electrodes in the motor area recorded neural activity, detected APs, calculated the instantaneous firing rate, and determined the movement velocity of the left and right wheels of the robot through linear decoding. At the same time, the computer monitors the left and right sensors of the mobile robot and encodes them as stimulation signals for the left and right stimulation electrodes (encoding sensor values as stimulation frequencies). In this way, the BNN can guide the mobile robot to perform obstacle avoidance tasks. The key to the stable obstacle avoidance of this system is that the sensory area and the motor area have a stable stimulus–response relationship. Obstacle avoidance can be achieved if an increase in the stimulation frequency of the sensory area can steadily induce an increase in the activity of the corresponding motor area. A follow-up study improved decoding strategies, which achieved better performance [38]. After that, in order to further improve the obstacle avoidance ability of this neurorobotic system, Tessadori et al. [41] modified the network structure of the BNN. They used a polydimethylsiloxane mask to divide the BNN on MEA into 2 modules connected by only 2 microchannels, therefore forming a modular BNN, which exhibited higher channel selectivity and further improved its obstacle avoidance ability.

In these studies, neural signals were recorded in real time and decoded to control the robot’s movements. However, in other studies, only the neural signals evoked by stimulation signals were used for robot control. DeMarse et al. [37] used an in vitro BNN to control the pitch and roll angles of an aircraft in a simulated environment so that it could maintain a level and straight flight. They used an MEA to connect the BNN with the simulated aircraft. Specifically, they chose 2 stimulating electrodes on the MEA to control pitch and roll angles, respectively. After each stimulation signal was applied, the recorded neural signals were calculated as the current flight vector, which was compared to the original one to produce a correction amount, thereby correcting the flight error. In another study, Warwick et al. [36] used an in vitro BNN to control a mobile robot Miabot for obstacle avoidance tasks. They first identified stable neural circuits in BNNs as the input–output electrode pairs, one of which was then selected to make decisions for robot motion. A complete closed-loop robot control loop was constructed: When the robot encounters an obstacle, it sends a signal to the input electrode to apply a stimulus signal, which causes the output electrode to detect a neural signal that guides the robot to turn and avoid the obstacle.

In addition to identifying specific input–output responses as sensory-motor mappings, by treating the entire BNN as a complex physical system, reservoir computing can be further used to control robots to perform specific tasks. In an early study, Pizzi et al. [110] cultured a human BNN on MEA to connect it with a mobile robot. The human BNN was formed by first seeding human fetal neural stem cells on MEA and then differentiating them by brain-derived neurotrophic factor. After that, 4 stimulus patterns were applied on the BNN, with each one symbolically representing the robot’s moving direction. The evoked neural activities were recorded through EAP recording and classified by an ANN to decode which stimulus was applied. The robot moved in that direction accordingly. This work demonstrated that in vitro human BNN presented a response specificity to different stimuli (mapping input stimuli to another signal space), which can then be classified by an ANN. This can be leveraged to build sensory-motor mappings between BNNs and robots. In a later research, the NTNU Cyborg project connected the BNN with a simulated robot through an MEA and utilized the BNN to perform reservoir computing. The recorded neural signals were passed to an ANN reader (output layer) to determine the motion state of the mobile robot (rotational speed of 2 independent driving wheels). At the same time, the sensor output of the robot is encoded and used to determine the stimulation signal (stimulation time) of the MEA stimulation electrode, which acts on the BNN through the input layer of the BNN, forming a closed loop. By incorporating the robot’s distance from the wall into the cost function and training the weights of the ANN reader based on a genetic algorithm, the mobile robot can be successfully guided to avoid hitting the wall. In another study, Yada et al. [43] used an in vitro BNN as the computational reservoir to guide a mobile robot to reach a target location. This study connected the BNN with an MEA and integrated the recorded signals into a time-varying output by a linear reader. The linear reader is trained on the basis of FORCE learning so that its time-varying output can be kept around a target constant. During training, in addition to the linear reader’s weights being updated in real time, the difference (error) between the reader’s output and the target constant is fed back to the BNN through optical stimulation with caged glutamate. By adjusting the illumination time, the error of the linear reader can be fed back to the BNN. After training, the weights of the linear readers remain unchanged, while their errors are still fed back to the BNN. The error was used to control a mobile robot to reach the desired target successfully.

### Network plasticity-dependent intelligent behaviors

Network plasticity refers to the change of synaptic strength of all neurons in a BNN induced by the neural activity in it. This change is thought to be a fundamental mechanism by which the brain is able to store memories, learn, understand, and adapt to the environment. The in vitro BNNs also showed network plasticity, and studies have revealed that they can present intelligent behaviors such as short-term memory and learning. These contents have already been covered in the Short-term memory and Learning subsections and, therefore, will not be reiterated here. In this section, we focus on how these mechanisms and the induced changes in neural activity of BNNs can be used to control robots.

**Table 2.** Representative references on network plasticity-dependent robot control.

Ref.	Recording/ stimulation	Encoding/decoding	Training	Robot tasks
[42]	EAP/electrical	Proportional coding basded on the relative position/binary coding based on motor areas’ firing frequency	If the paddle misses the ball, apply a negative feedback; apply a positive one otherwise.	Playing the video game “Pong”
[54]	EAP/electrical	Fixed probe stimuli/binary coding based on motor areas’ firing frequency	If the robot moves in the wrong direction, apply a corrective feedback; apply no feedback otherwise.	Tracking an immobile object
[112]	EAP/electrical	Fixed probe stimuli/population coding based on the entire BNN’s firing frequency	If the robot moves to an undesired area, apply a corrective feedback; apply no feedback otherwise.	Remaining in a user-defined area
[113]	EAP/electrical	Fixed probe stimuli/population coding based on the entire BNN’s firing frequency	If the robot moves in the wrong direction, apply a corrective feedback; apply no feedback otherwise.	Moving to and then remaining in a user-defined area
[114]	EAP/electrical	Fixed probe stimuli/population coding based on the entire BNN’s firing frequency	If the robot moves in the wrong direction, apply a negative feedback; apply a positive one otherwise.	Moving to and then remaining in a user-defined area
[115]	EAP/electrical	Fixed probe stimuli/population coding based on the entire BNN’s firing frequency	If the robot moves in the wrong direction, apply a negative feedback; apply a positive one otherwise.	Moving in a prechosen direction
[116]	EAP/electrical	Proportional coding with threshold/ population coding based on motor areas’ firing frequency	If the robot meets obstacles, stop applying feedback stimuli; apply feedback stimuli otherwise.	Obstacle avoidance

Among the researches on embodying in vitro BNNs with robots, some only utilized their computing capacity to construct specific sensory-motor responses to achieve robot control, that is, the computing capacity-dependent robot control introduced in the Computing capacity-dependent robot control subsection. On the other hand, other studies realized robot control through the changes of BNNs’ synaptic strength and/or corresponding neural activity caused by the stimulation input, that is, the network plasticity-dependent robot control to be introduced here. A list of representative research on network plasticity-dependent robot control is shown in Table 2.

In an early study, Bakkum et al. [112] used an MEA to interconnect an in vitro BNN with a painting robot to control the robot to fill a square area. The robot can draw on a flat surface [112]. A fixed probe stimulus signal applied via a stimulating electrode induced the BNN to generate evoked neural activity, which was recorded and mapped to a 2D vector CA (the center of neural activity) using population coding. The position of the pen tip of the painting robot was then determined accordingly. Every 5 min, the painting results were evaluated to determine whether to apply additional stimulus on the BNN. If the painting result is expected (most of the newly added patterns in the last 5 min are in the square area), then no additional stimulus is added; otherwise, a patterned background stimulation is applied according to its deviation, thereby correcting the CA. Because of the spontaneous activity of the neural network, the CA will gradually shift over time, and the purpose of this study is to use the network plasticity of the neural network to keep the CA always within a certain range by applying feedback electrical stimulation signals. In a follow-up study, the authors utilized the neurorobotic system to achieve a goal-directed behavior [113]. Unlike in the former study [112], where CA was mapped to the absolute coordinates of the robot’s next step, in this study, the CA was mapped to the relative displacement of the robot’s next step. It demonstrated that the use of appropriate electrical stimulation can selectively alter the internal synaptic connectivity of the BNN, thereby enabling its neural activity output (CA) to fall within the desired quadrant. Bakkum et al. [114] improved on the strategy of choosing stimulation patterns. Unlike in previous studies, where the appropriate stimulation pattern was chosen on the basis of experimental statistical results, they employed an adaptive algorithm to learn stimulus application strategies online, which optimally selects the appropriate stimulus by adjusting the probability of selection of a randomly applied feedback stimulus based on the performance of the robot. All the above studies demonstrated that the appropriate choice of stimulation patterns can produce desired changes in the functional connectivity and neural activity of the in vitro BNN.

These studies took advantage of the mechanism of spike-timing-dependent plasticity, which states that when the pre- and postsynaptic neurons produce APs in sequence in a short time, the synapse will be strengthened; otherwise, the synapse will be weakened [115]. Therefore, any stimulation can cause changes in synaptic strength (enhanced or weakened), thereby altering the CA. However, this method suffers from relatively random changes in the network synaptic connectivity and the output CA due to the complexity and time-varying network structure in BNN. As a result, the mapping from a specific stimulation pattern to a specific output CA is time-varying and therefore hard to be depicted, which makes it necessary to identify and correct this mapping in real time along with the experiment.

In another study, Li et al. [54] used a different approach to realizing a target-finding task. In this study, similar to previous research on computing capacity-based robot control, the authors constructed 2 sensory-motor-specific circuits to determine the robot’s motor control signals based on input sensory signals, but the input stimulus signal is constant, and the robot’s sensory signal is fed back to another additional stimulus that causes synapses to change, thereby changing the response of the sensory-motor circuit, which, in turn, affects the robot’s control signals.

As mentioned before, in a pioneering work, Shahaf and Marom [24] found the in vitro BNNs can perform supervised learning through closed-loop training. Masumori et al. [116] took a step further by connecting this BNN with a mobile robot, and the BNN’s firing patterns were therefore mapped to the robot’s behavior. Likewise, each time the robot exhibited a wall-avoidance behavior, stimulation was stopped. Through this supervised learning process, the mobile robot “learned” to actively avoid walls each time it meets them.

In these studies, the sensor signals of the robot were fed back and encoded as stimulus signals that alter the network connectivity (synaptic strength) of the BNN, and then the state or state changes of this network connectivity are decoded to control the robot’s movements. Therefore, determining the mapping from stimulation patterns to changes in network connectivity is critical for the success of robot tasks. However, this mapping varies by BNN, robot task, encoding strategy, decoding strategy, and changes over time, making it difficult to determine. For this problem, the recently proposed free energy principle offers a potential and universal solution. Kagan et al. [42] trained an in vitro BNN based on the free energy principle to play a video game “Pong”. In the “Pong” game, there are 3 walls and a “paddle” that can move up and down. A small ball will bounce back and forth between the wall and the paddle. BNN needs to control the paddle to move up and down, to hit the moving ball. The researchers constructed a sensory-motor pathway to connect the BNN in the dish with the “paddle” (the robot) in the “Pong” game environment through a high-resolution MEA. The relative positional relationship between the ball and the paddle (up or down) is encoded as a stimulus signal and applied to electrodes in the sensory area of the BNN in real time. Therefore, the BNN in the dish can “know” the current position of the ball relative to the paddle in real time. At the same time, the neural activity of the 2 motor areas is recorded in real time, and their intensity relationships are compared to determine the direction of the paddle’s movement at the next moment. To train the BNN to play “Pong”, the researchers designed a supervised learning method. Its specific strategy is as follows. Each time the paddle misses the ball, a negative feedback (a randomly selected new electrical stimulation signal) is applied to the sensory area; and every time the paddle successfully receives a ball, a positive feedback (a fixed electrical stimulation signal) is applied to the sensory area. The free energy principle holds that any self-organizing living system (including in vitro BNNs) will actively build a model that predicts the environmental stimulus it receives and adjust its model or actively act on the environment to reduce its prediction (predictive stimulus) and observation (actual stimulus) error. This theory was validated in the in vivo BNNs [83]. According to this theory, when the paddle misses the ball and a random electrical stimulation signal is applied to the sensory area of BNN, since the signal is unpredictable, the error between prediction and observation will be very large. In order to reduce this error, the BNN will actively adjust its model (change the synaptic strength of its network through network plasticity) to avoid the appearance of this stimulus signal, that is, BNN tends to actively manipulate the paddle to contact the ball. Therefore, the selection of positive and negative feedback signals here does not need to depend on mapping from certain stimulation patterns to changes in network connectivity. In theory, any electrical stimulation signal, as long as it is applied fixedly and repeatedly, can be used as positive feedback; and any electrical stimulation signal, as long as it is applied randomly, can be used as negative feedback. This theory provides great convenience for training the in vitro BNNs.

## Trends and Challenges

### Fabricating BNNs in 3D

One important feature of the brain is the 3D distribution of its neurons. To reproduce this characteristic, many researchers have begun to explore the fabrication of 3D in vitro BNNs [117–119]. Because of the 3D distribution, the number of neurons and the number of synapses in the BNN can be greatly increased. As a sufficient number of neurons and synapses underlies the birth of intelligence [24], the research on cultivating 3D BNNs will undoubtedly attract more attention. However, it also faces many challenges, including the following: (a) Current biocompatible materials for culturing 3D BNNs are inferior to extracellular matrix gels in the brain in many respects, so they remain to be developed and improved [120]; (b) the supply of nutrients limits the size of 3D BNN, so 3D vascularization techniques for large-size 3D BNNs need to be investigated [121,122]; and (c) the development of the brain is a bottom-up self-organization process, which can form extremely complex biological structures based on simple organization principles [5]. This process outperforms all the state-of-the-art biofabrication methods, so more complex 3D BNNs require more advanced biofabrication methods. Moreover, along with constructing 3D in vitro BNNs, techniques for recording (e.g., 3D MEAs [123,124]) and stimulating (e.g., focused ultrasound [125,126]) neurons precisely in 3D BNNs are attracting increasing attention.

### Custom-designing BNNs with desired structural and functional connectivity

Another important feature of the brain is the specificity of its neuronal distribution and synaptic connections. The neuronal distribution is not uniform but modular [82]. In addition, the synaptic connections are not randomly connected but with specific connection directions in the neurogenesis process. Therefore, many studies have begun to realize BNNs with specific structural and functional connectivity in vitro [127–130]. Commonly used methods are microfluidic technology and microcontact printing. Currently, the unidirectional connection between single neurons [105], the unidirectional connection between 2 neuronal modules (nerve groups) [28,102–104], and the directed connection between 3 neuronal modules can be realized [76]. However, to achieve large-scale 2D BNNs with desired structural and functional connectivity, these challenges still need to be addressed: (a) Scalability of current methods needs to be improved; and (b) neuronal seeding itself brings uncertainty, so the investigation of artificial environmental cues for neuronal self-organization is important.

### Improving BNN–robot bidirectional connection

In addition to the above inherent characteristics of the brain, the bidirectional connection between the brain and the body is another important factor in the brain’s ability to understand and adapt to the external environment [34]. This connection features low latency, stability, long term, and large channel throughput. In order to build a brain model in vitro and endow it with the conditions to generate intelligence, the realization of bidirectional connections between in vitro BNNs and robots with these characteristics has gradually become a research hotspot [43,111,131]. However, the main challenges are the following: (a) The channel capacity of the bidirectional connection between BNNs and the robot needs to be improved, in which complementary metal–oxide semiconductor chips are being tested for neural recording with higher spatial resolution [116,132,133]; (b) various recording and stimulation methods have their own advantages and disadvantages, so different recording and stimulation methods need to be integrated to achieve more advanced interaction between BNNs and robots [85,131]; and (c) more efficient neural encoding and decoding methods need to be studied.

### Training the robot-embodied BNN

Finally, increasing studies directly use in vitro BNNs as the “brain” of robots to build robots for control and decision-making based on biological intelligence [44,54]. However, it is not enough to construct an in vitro biological brain with the potential to express intelligence and to achieve a stable bidirectional connection with a robot. Training the BNN to make it understand and perform specific tasks by applying the appropriate external stimuli is also essential [24,58]. Therefore, how to train the in vitro BNNs to perform specific robot tasks has received increasing attention [42,116]. The main challenges it faces are as follows: (a) Since BNNs are only composed of neurons and lack the participation of various neuromodulators, various training methods for animals are difficult to be transplanted into the training of BNNs, which increases the difficulty of training BNNs; (b) the limitations of biological intelligence itself. While a monkey can be trained to ride a bicycle, it is much harder to do when it comes to tasks requiring higher-level thought processes, such as playing Go. (c) Stable communication is the basis of training, but it is difficult to find a suitable way to communicate with the BNNs since we do not know the right coding mechanism of interneuronal and intranetwork communication in BNNs yet.

## Conclusion

Dissociated neurons can form an in vitro BNN and hence hold the potential to present biological intelligence with the help of a robot body. In this review, we introduce the theoretical background for this to happen, summarize the mainstream ways of interconnecting in vitro BNNs with robots, detail the intelligent behaviors presented in in vitro BNNs with a focus on those related to robot intelligence, and present some promising developmental trends and their corresponding challenges. Combining in vitro BNNs with robots is an interdisciplinary field crossing neuroscience, bioengineering, robotics, and microelectromechanical systems, among others; so with the flourishing developments of these domains, it will definitely witness increasing encouraging breakthroughs and bring us closer to biological brain-based robot intelligence.

## Data Availability

The original data supporting this review are from previously reported studies and datasets, which have been cited. The processed data are available from the corresponding author upon request.
